# Supramolecular Design Strategy of a Water-Soluble Diphenylguanidine-Cyclodextrin Polymer Inclusion Complex

**DOI:** 10.3390/molecules27206919

**Published:** 2022-10-15

**Authors:** Junqiang Guo, Liwei Lin, Yuping Wang, Wang Zhang, Guowang Diao, Yuanzhe Piao

**Affiliations:** 1 School of Chemistry and Chemical Engineering, Yangzhou University, Yangzhou 225002, China; 2 Department of Applied Bioengineering, Graduate School of Convergence Science and Technology, Seoul National University, Seoul 08826, Korea; 3 Research Institute for Convergence Science, Seoul National University, Seoul 08826, Korea; 4 Advanced Institutes of Convergence Technology, Suwon 16229, Korea

**Keywords:** diphenylguanidine, cyclodextrin polymer, inclusion complex, solubility, characterization

## Abstract

Diphenylguanidine (DPG) is a widely used secondary accelerator for the vulcanization of natural rubber (NR) latex. However, its low water solubility and high toxicity limit its use in high-end NR products. In this study, a water-soluble inclusion complex of DPG and a *β*-cyclodextrin polymer (*β*-CDP), termed DPG-*β*-CDP, was prepared through supramolecular interactions and characterized using Fourier-transform infrared spectroscopy, ^1^H NMR, scanning electron microscopy, and UV-vis spectroscopy techniques. In comparison with that of DPG, the water solubility of DPG-*β*-CDP was greatly enhanced because of the water-soluble host molecule. The molar ratio of DPG to the CD unit in *β*-CDP was determined to be 1:1. At 25 °C, the binding constant of DPG-*β*-CDP was found to be 9.2 × 10^5^ L/mol by UV-vis spectroscopy. The proposed method for forming inclusion complexes with high potential for use as water-soluble vulcanization accelerators is promising.

## 1. Introduction

Diphenylguanidine (DPG) is widely used as an important accelerator in natural rubber vulcanization process that can improve the mechanical properties of rubber products [1,2,3]. In applications of the rubber industry, energy-intensive ball milling is generally required to maintain the DPG dispersion in liquid phase due to its low solubility in water [4]. In addition, DPG, as a dermatological sensitizer and allergen, exposures during production or contamination of the final product may lead to allergic contact dermatitis, reproductive toxicology, and environmental contamination [5,6]. These issues significantly limit the utilization of DPG.

Cyclodextrin polymers (CDPs) are an important class of host molecules that encapsulate various types of guest molecules [7,8,9]. They have a cone structure of cyclodextrin (CD) consisting of a hydrophobic cavity and hydrophilic exterior in polymer chains [10]. Therefore, non-polar guest molecules with appropriate molecular sizes can be adsorbed into the hydrophobic cavity and form inclusion complexes, which are easily dispersed in water owing to their external hydrophilicity [11]. Encapsulating a hydrophobic molecule in CDP has several benefits, including improved water solubility, increased water dispersibility, and a controlled release profile [12]. CDs are usually classified into three types, namely, α-, *β*-, and γ-cyclodextrins, of which *β*-CD has a moderate cavity size and thus the widest application range [13]. CDP can improve the water solubility and biological activity of CD, which exhibits structural rigidity and low solubility. In recent years, CDP has been reported to retain the inclusion properties of CD monomers, improve the water solubility of CD, and show good stability and chemical tenability [14,15,16].

There are numerous low-cost methods for the preparation of *β*-cyclodextrin polymer (*β*-CDP) inclusion complexes, including grinding, freeze-drying, and ball milling [17]. In this study, grinding was used to prepare DPG-*β*-CD polymer inclusion complexes. The successful synthesis of the inclusion complex was verified using characterization methods such as Fourier-transform infrared (FTIR) spectroscopy, X-ray diffraction (XRD), and ^1^H NMR spectroscopy. The host-guest interaction between DPG and *β*-CDP in the inclusion complex was studied by the UV-vis spectroscopy, and the solubilizing effect of DPG-*β*-CDP on DPG was studied using the phase solubility method.

## 2. Results

**Figure 1:**
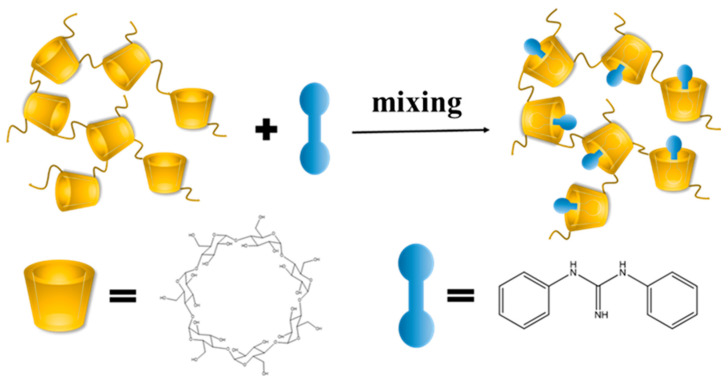
Schematic illustration of procedure for DPG-*β*-CDP.

**Figure 2:**
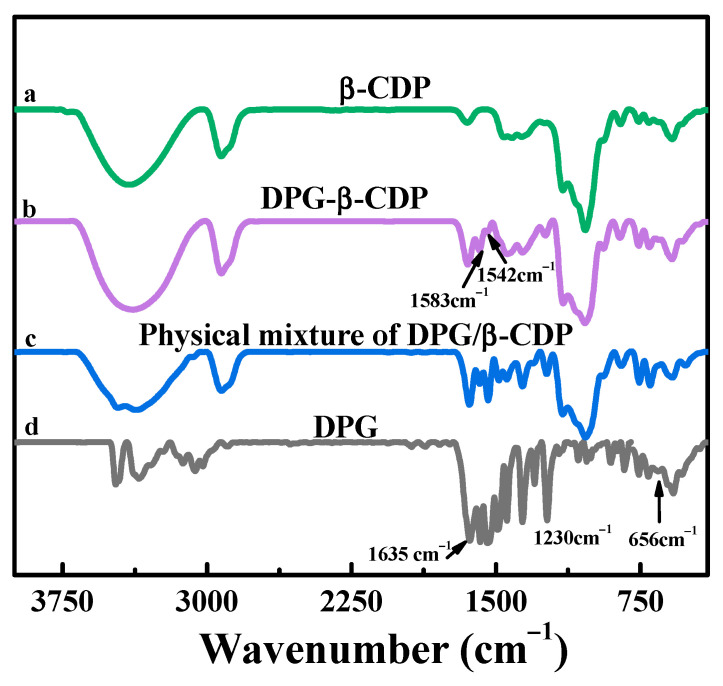
FT-IR spectra of DPG-*β*-CDP and comparison with other substances.

Figure 1 illustrates the synthesis process for DPG-*β*-CDP. The infrared spectra of *β*-CDP, DPG, the inclusion complex of DPG-*β*-CDP, and the physical mixture of DPG/*β*-CDP are listed in Figure 2. The stretching vibrations of -OH, -CH_2_ and C-O-C were observed at 3394, 2929 and 1032 cm^−1^ for *β*-CDP, respectively. DPG primarily exhibits the stretching vibrations of C=N (1635 cm^−1^) and C-N (1234 cm^−1^) as well as the out-of plane bending vibration of N-H (656 cm^−1^) [18]. The infrared spectrum of the physical mixture of DPG/*β*-CDP was a superposition of the DPG and *β*-CDP spectra. In the inclusion complex, the characteristic peaks of the N−H stretching vibrations of DPG at 3471 and 3350 cm^−1^ disappeared because of the shielding effect in the *β*-CDP cavity, whereas those of *β*-CDP were retained. The characteristic DPG peaks were observed only at 1583 (benzene ring skeleton vibration) and 1542 cm^−1^ (C=N stretching vibration), and both peaks exhibited a slight red shift. This result indicates that the DPG molecules entered the hydrophobic *β*-CDP cavities via host-guest interactions, suggesting the formation of DPG-*β*-CDP.

**Figure 3:**
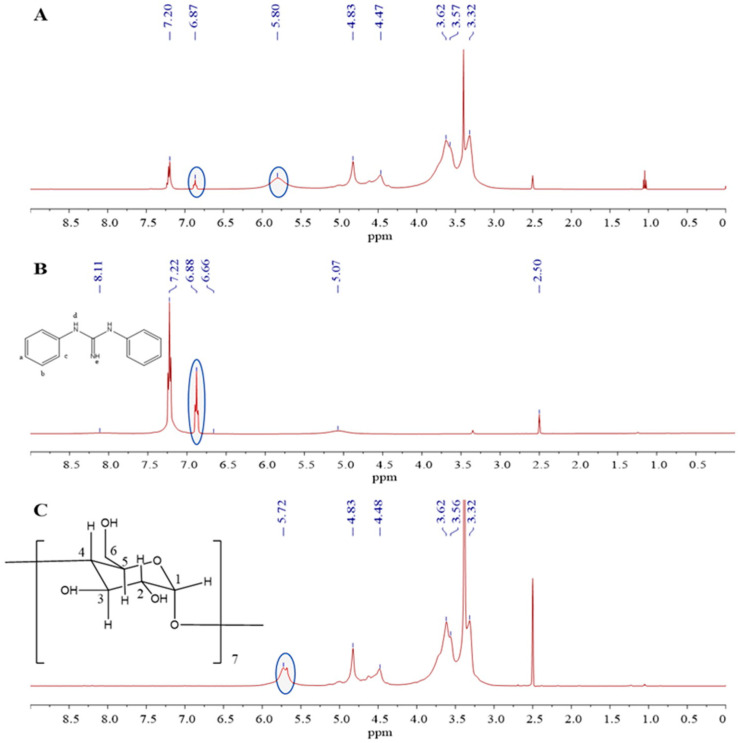
^1^H NMR spectra of (**A**) DPG-*β*-CDP, (**B**) DPG, and (**C**) *β*-CDP.

The ^1^H NMR spectroscopy is an effective method for investigating supramolecular interactions [19,20,21]. The ^1^H NMR spectra of the inclusion complex, DPG, and *β*-CDP are shown in Figure 3. In addition, the chemical shifts of DPG-*β*-CDP are listed in Table 1. After DPG-*β*-CDP formation, the OH-2 and OH-6 chemical shifts of the *β*-CD changed from 4.48 and 5.80 ppm to 4.47 and 5.72 ppm, respectively (Table 1). The proton displacements of H-a and H-b, c of DPG in the inclusion complex changed from 6.88 and 7.22 ppm to 6.87 and 7.20 ppm, respectively. The relatively small change in the chemical shift showed the supramolecular interactions between DPG and *β*-CDP and indicated that DPG molecules successfully entered CD cavities of *β*-CDP [22].

The stoichiometric ratio of the host and guest molecules using the integrated peak ratio of host/guest molecule was calculated in our previous study [14]. According to the results in Figure 3A and Table 1, the OH-2 of *β*-CDP and H-a of DPG do not overlap with other peaks. The area of the OH-2 peak of the CD unit in polymer chains was marked as 1, and the integrated peak of DPG was found to be 0.21; therefore, the integrated peak ratio of DPG to the CD unit in *β*-CDP is 1.36. Therefore, the molar ratio of DPG to the CD unit in *β*-CDP was calculated to be approximately 1:1 based on the above results.

**Figure 4:**
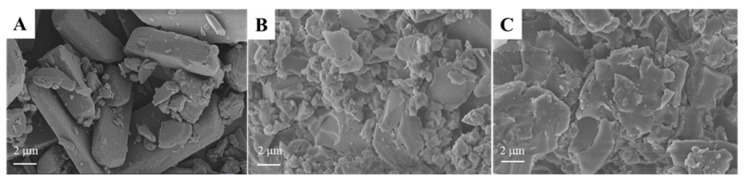
SEM images of (**A**) DPG, (**B**) *β*-CDP, and (**C**) DPG-*β*-CDP.

The SEM images of DPG, *β*-CDP, and the DPG-*β*-CDP inclusion complex are shown in Figure 4. The surface morphology of DPG is rod-like with irregular surfaces, whereas *β*-CDP has a random sheet-like structure as an amorphous polymer. However, in the SEM image of DPG-*β*-CDP (Figure 4C), the DPG rod-like structure disappeared, and only irregularly sized sheets were observed, which indicates that the cavities of *β*-CDP were saturated with DPG. These morphological changes suggest the occurrence of supramolecular interactions due to morphological variation of DPG-*β*-CDP.

**Figure 5:**
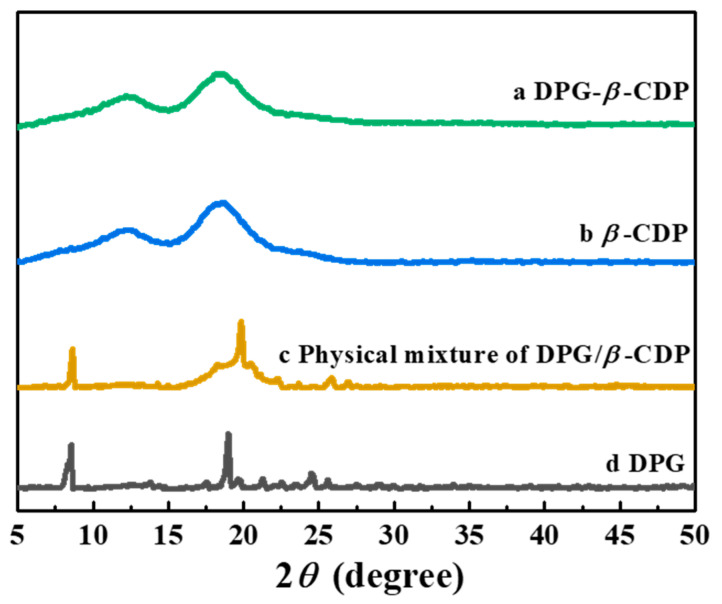
XRD patterns of DPG-*β*-CDP, DPG, and *β*-CDP, the physical mixture of DPG and *β*-CDP.

As shown in Figure 5, the DPG-*β*-CDP, DPG, and *β*-CDP crystal structures were examined using XRD tests. DPG has a good crystal structure with many sharp diffraction peaks at 8.53, 13.80, 17.51, 18.98, 22.61 and 24.50°. The *β*-CDP diffraction pattern showed no significant crystal peaks, which indicates that *β*-CDP had an amorphous state. Furthermore, both amorphous states of *β*-CDP and multiple DPG diffraction peaks were observed in Figure 5 c. This indicated that there was no new crystal formation in the physical mixtures [16]. The diffraction pattern of the DPG-*β*-CDP inclusion complex was similar to that of *β*-CDP, in which the DPG peaks were indistinct. Since DPG was encapsulated in the *β*-CDP CD cavity, the crystal properties of DPG were covered, which further verified formation of DPG-*β*-CDP successfully.

**Figure 6:**
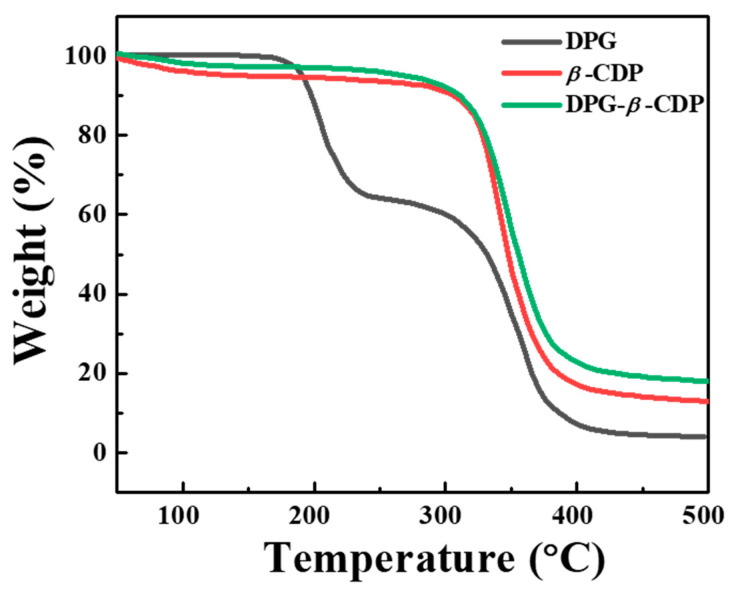
TGA curves of DPG-*β*-CDP, DPG, and *β*-CDP.

To test the thermal stability, the TGA result of DPG-*β*-CDP inclusion complexes from 30 to 500 °C are shown in Figure 6. The TGA curve of the inclusion complex is similar to that of *β*-CDP, which shows that water loss occurs between 30 and 100 °C. However, the quantity of water evaporated from inclusion complexes was less than that from host polymers, which was due to the replacement of a portion of the bound water by DPG molecules in the inclusion complex. The DPG-*β*-CDP inclusion complex begins to decompose at approximately 300–500 °C, which is the same as the temperature range for *β*-CDP decomposition. Moreover, the pure DPG began to lose weight at 166 °C, which is significantly lower than the temperature for DPG-*β*-CDP, indicating that the inclusion complex of DPG with *β*-CDP can effectively improve the thermal stability of DPG. The decomposition temperature change is an important factor indicating the formation of DPG-*β*-CDP.

**Figure 7:**
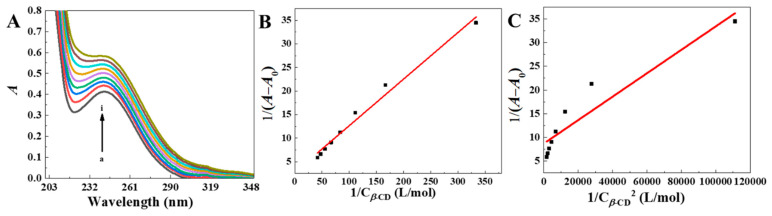
(**A**) At 25 °C, the UV-vis spectra of the 3 × 10^−5^ mol∙L^−1^ DPG solution with different concentrations of *β*-CDP (C_*β*-CD_: *β*-CD unit in *β*-CDP, mmol/L): (a) 0, (b) 3, (c) 6, (d) 9, (e) 12, (f) 15, (g) 18, (h) 21, and (i) 24. (**B**) Plot of $\frac{1}{A-A_{0}}$ versus $\frac{1}{C_{\beta \text{-CD}}}$. (**C**) Plot of $\frac{1}{A-A_{0}}$ versus $\frac{1}{C_{\beta \text{-CD}}^{2}}$; *C_β_*_-CD_ represents the CD unit concentration in *β*-CDP.

To estimate the host–guest interactions, the UV-vis spectra of DPG solutions containing different concentrations of *β*-CDP with ethylene glycol as the co-solvent were obtained (Figure 7A). The DPG absorption intensity raised with *β*-CDP concentration, indicating the host-guest interactions. It is assumed that the molar ratio of DPG to the *β*-CD unit in *β*-CDP is 1:1 or 1:2; the following equations were used for the calculations [16,23]:

(1) $$\frac{1}{\left[A-A_{0}\right]}=\frac{1}{\left[\Delta \varepsilon \right]}+\frac{1}{K_{a}{\left[G\right]}_{0}\left[\Delta \varepsilon \right]\left[H\right]}$$

(2) $$\frac{1}{\left[A-A_{0}\right]}=\frac{1}{\left[\Delta \varepsilon \right]}+\frac{1}{K_{a}{\left[G\right]}_{0}\left[\Delta \varepsilon \right]{\left[H\right]}^{2}}$$

(3) $$\Delta \varepsilon =\varepsilon_{HG}-\varepsilon_{H}-\varepsilon_{G}$$

where [*G*]_0_ is the initial concentration of the guest molecule (DPG), mol/L; [*H*] is the concentration of the host molecule (CD unit in *β*-CDP), mol/L; *A* is the DPG absorbance after adding *β*-CDP; *A*_0_ is the absorbance of a pure DPG solution; [*A* − *A*_0_] is the absorbance change (i.e., the absorbance of the inclusion complex); *ε_H_*, *ε_G_*, and *ε_HG_* are the molar absorption coefficients of *H*, *G* and *HG*, respectively; and *K_a_* is the binding constant of the host and guest molecules, L/mol.

We assumed that the inclusion molar ratio of DPG to CD unit in polymer chains was 1:1 or 1:2. According to Equations (1) and (2), there are linear fitting curves illustrating the phenomenon in Figure 7B,C. When the molar ratio was assumed to be 1:1, the linear correlation in Figure 7B was higher (R^2^ = 0.986) than that (R^2^ = 0.876) in Figure 7C. This indicates that CD structural units in DPG-*β*-CDP is closely packed with DPG at a molar ratio of 1:1. The *K_a_* of DPG-*β*-CDP was calculated to be 9.2 × 10^5^ L/mol, indicating that *β*-CDP can form a relatively stable inclusion complex with DPG. Compared with DPG-CD (*K_a_* = 8.6 × 10^5^ L/mol) [16], the larger diffusion coefficient of DPG-*β*-CDP suggested that *β*-CDP formed an inclusion complex of DPG more easily than CD monomer, which showed that the polymer chain had stronger intermolecular interactions.

**Figure 8:**
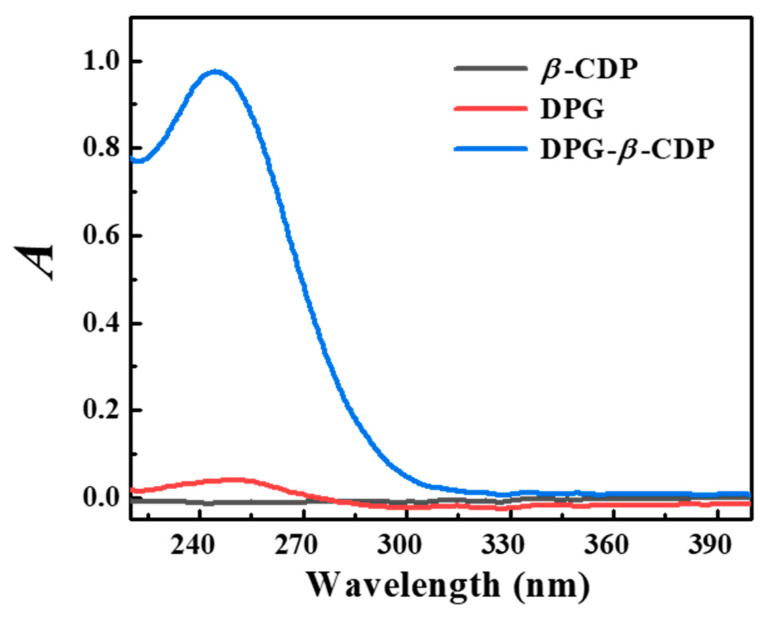
UV-vis spectra of excess DPG, *β*-CDP, and DPG-*β*-CDP aqueous solutions.

**Figure 9:**
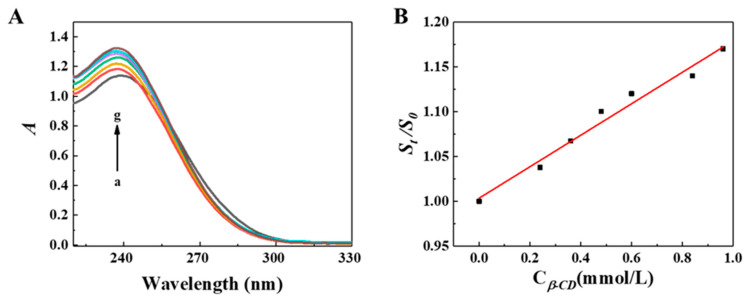
(**A**) UV-vis spectra of excess DPG aqueous solution and *β*-CDP with different concentrations (*C_β_*_-CD_: *β*-CD unit in *β*-CDP, mmol/L): 0, 0.43, 0.57, 0.86, 1.14, 1.71, 2.56, 3.42 and 4.28. (**B**) Dissolution curve of DPG in the *β*-CDP aqueous solution.

As shown in Figure 8, *β*-CDP has no UV absorption peak from 220 to 401 nm, which does not affect the inclusion complex, whereas DPG in DPG-*β*-CDP has much higher absorption peaks at 243 nm compared with those of DPG. The DPG solubility was significantly improved by complexation with *β*-CDP. The solubility of the DPG-*β*-CDP inclusion complex was 0.073 mmol/L, while that of DPG was 0.0044 mmol/L, representing a 16.7-fold increase. The solubilization effect of DPG in different *β*-CDP concentrations is illustrated in Figure 9, and the DPG solubility in water increased with an increase in *β*-CDP concentration. This linear relationship can be expressed using equation 4 [16,24]:

(4) $$\frac{S_{t}}{S_{0}}=1+K_{C}C_{0}$$

where *S_t_* represents the DPG concentration in solutions with different *β*-CDP concentrations (mmol/L); *S*_0_ represents the intrinsic DPG concentration (mmol/L); *C*_0_ represents the initial *β*-CDP concentration (mmol/L); and *K_C_* represents the solubilization coefficient (L/mol), which was evaluated the solubilizing ability of *β*-CDP to DPG.

According to Equation (4), the *K_C_* of *β*-CDP to DPG can be obtained as 175 L/mol, indicating that *β*-CDP has a good solubilization effect on DPG. Additionally, the linear correlation result (R^2^ = 0.977) also confirmed that DPG and the *β*-CD unit in *β*-CDP had a molar ratio of 1:1 in the inclusion complexes.

## 3. Materials and Methods

1,3-DPG; *β*-CD; sodium hydroxide (NaOH); hydrochloric acid (HCl); and other reagents were purchased from Sigma-Aldrich (Shanghai, China). They were of analytical grade without additional purification. A water purification system was used to obtain disinfected twice-distilled water. *β*-CDP was synthesized under a previously reported protocol [14].

Figure 1 illustrates the synthesis process for DPG-*β*-CDP. In a typical synthesis process, the inclusion complex was prepared by dissolving 3.5 g of CDP and 0.75 g of DPG in 50 mL of water and stirring the mixture for 24 h at 25 °C. An off-white solution containing DPG-*β*-CDP was subsequently obtained. Then the solution was filtered to remove insoluble DPG. The product was then rotary-evaporated using pressure distillation. At last, it was dried in a vacuum oven at 70 °C for 30 h. The saturated solubility of samples in water was measured at 25 °C in accordance with a previously reported approach [25].

Scanning electron microscopy (SEM, Zeiss Supra-55VP, ZEISS, Jena, Germany) was used to observe the morphologies of the samples. The composites were analyzed using an FTIR spectrophotometer (Antaris II). ^1^H NMR spectra were recorded on a 600 MHz Bruker spectrometer (Ascend, 600, Bruker, Karlsruhe, Germany) at 303.1 K in DMSO-d6. XRD data were obtained using a D8 high-speed powder diffractometer (Bruker, Karlsruhe, Germany), and phase analysis of the Raman spectra was performed. Thermogravimetric analysis (TGA) measurements were conducted with a TG209F3 Tarsus (NETZSCH, Selb, Germany) from room temperature to 500 °C at a heating rate of 10 °C·min^−1^ in a dynamic N_2_ atmosphere. The UV-vis spectrum was recorded on a UV-2550 double-beam spectrophotometer (Shimadzu, Kyoto, Japan) equipped with a stoppered quartz cell with a 1.0 cm optical path length.

## 4. Conclusions

In this study, we overcame the challenges of insolubility of DPG and proposed a design strategy for the inclusion complex of DPG-*β*-CDP. Through host–guest interactions, *β*-CDP endows DPG with greatly enhanced water solubility and dispersibility. It was confirmed that the host–guest molar ratio of the DPG-*β*-CDP inclusion complex was 1:1 and the binding constant was obtained by UV-vis spectroscopy. These results proved that *β*-CDP inclusion strategy would be a promising road to improve the application of DPG.

## Figures and Tables

**Table 1:** Chemical shift *δ* and Δ*δ* of protons of DPG, *β*-CDP, and DPG-*β*-CDP.

Title 1	OH-2	OH-6	H-a	H-b, c
DPG	-	-	6.88	7.22
*β*-CDP	5.72	4.48	-	-
DPG-*β*-CDP	5.80	4.47	6.87	7.20
Δ*δ*	0.08	−0.01	−0.01	−0.02

## Data Availability

Not applicable.

## Sample Availability

Samples of the compounds are available from the authors upon a reasonable request.

## References

[1] Hernández Santana M., den Brabander M., García S., van der Zwaag S. (2018). Routes to Make Natural Rubber Heal: A Review. Polym. Rev..

[2] Griebel J.J., Glass R.S., Char K., Pyun J. (2016). Polymerizations with elemental sulfur: A novel route to high sulfur content polymers for sustainability, energy and defense. Prog. Polym. Sci..

[3] Heideman G., Datta R.N., Noordermeer J.W.M., van Baarle B. (2004). Activators in Accelerated Sulfur Vulcanization. Rubber Chem. Technol..

[4] Akiba M., Hashim A.S. (1997). Vulcanization and crosslinking in elastomers. Prog. Polym. Sci..

[5] Sieira B.J., Montes R., Touffet A., Rodil R., Cela R., Gallard H., Quintana J.B. (2020). Chlorination and bromination of 1,3-diphenylguanidine and 1,3-di-o-tolylguanidine: Kinetics, transformation products and toxicity assessment. J. Hazard. Mater..

[6] Peter K.T., Tian Z., Wu C., Lin P., White S., Du B., McIntyre J.K., Scholz N.L., Kolodziej E.P. (2018). Using High-Resolution Mass Spectrometry to Identify Organic Contaminants Linked to Urban Stormwater Mortality Syndrome in Coho Salmon. Environ. Sci. Technol..

[7] Liu Y., Lin T., Cheng C., Wang Q., Lin S., Liu C., Han X. (2021). Research Progress on Synthesis and Application of Cyclodextrin Polymers. Molecules.

[8] Jansook P., Ogawa N., Loftsson T. (2018). Cyclodextrins: Structure, physicochemical properties and pharmaceutical applications. Int. J. Pharm..

[9] Yu H., Lin H., Xie Y., Qu M., Jiang M., Shi J., Hong H., Xu H., Li L., Liao G. (2022). MUC1 vaccines using β-cyclodextrin grafted chitosan (CS-g-CD) as carrier via host-guest interaction elicit robust immune responses. Chin. Chem. Lett..

[10] Crini G. (2014). Review: A History of Cyclodextrins. Chem. Rev..

[11] Celebioglu A., Saporito A.F., Uyar T. (2022). Green Electrospinning of Chitosan/Pectin Nanofibrous Films by the Incorporation of Cyclodextrin/Curcumin Inclusion Complexes: pH-Responsive Release and Hydrogel Features. ACS Sustain. Chem. Eng..

[12] Yao H., Wu M., Lin L., Wu Z., Bae M., Park S., Wang S., Zhang W., Gao J., Wang D. (2022). Design strategies for adhesive hydrogels with natural antibacterial agents as wound dressings: Status and trends. Mater. Today Bio..

[13] Topuz F., Uyar T. (2022). Advances in the development of cyclodextrin-based nanogels/microgels for biomedical applications: Drug delivery and beyond. Carbohydr. Polym..

[14] Zhang W., Xiao P., Lin L., Guo F., Wang Q., Piao Y., Diao G. (2022). Study of a water-soluble supramolecular complex of curcumin and β-cyclodextrin polymer with electrochemical property and potential anti-cancer activity. Chin. Chem. Lett..

[15] Liu Z., Ye L., Xi J., Wang J., Feng Z.-G. (2021). Cyclodextrin polymers: Structure, synthesis, and use as drug carriers. Prog. Polym. Sci..

[16] Zhang W., Lin L., Guo J., Wu M., Park S., Yao H., Paek S.H., Diao G., Piao Y. (2022). Design Strategy for Vulcanization Accelerator of Diphenylguanidine/Cyclodextrin Inclusion Complex for Natural Rubber Latex Foam with Enhancing Performance. Research.

[17] Zhao B., Jiang L., Jia Q. (2022). Advances in cyclodextrin polymers adsorbents for separation and enrichment: Classification, mechanism and applications. Chin. Chem. Lett..

[18] Li W., Zhang H., Yu C., Li T., Zhang X., Xiong C., Wang T. (2021). Efficient and Spectrally Stable Blue Light-Emitting Diodes Based on Diphenylguanidine Bromide Passivated Mixed-Halide Perovskites. ACS Appl. Electron. Mater..

[19] Guo F., Guo J., Zheng Z., Xia T., Chishti A.N., Lin L., Zhang W., Diao G. (2022). Polymerization of pyrrole induced by pillar[5]arene functionalized graphene for supercapacitor electrode. Chin. Chem. Lett..

[20] Wang Y., Wang D., Wang J., Wang C., Wang J., Ding Y., Yao Y. (2022). Pillar[5]arene-derived covalent organic materials with pre-encoded molecular recognition for targeted and synergistic cancer photo- and chemotherapy. Chem. Commun..

[21] Wang Q., Fan J., Bian X., Yao H., Yuan X., Han Y., Yan C. (2022). A microenvironment sensitive pillar[5]arene-based fluorescent probe for cell imaging and drug delivery. Chin. Chem. Lett..

[22] Cao Y., Chen Y., Zhang Z., Wang J., Yuan X., Zhao Q., Ding Y., Yao Y. (2021). CO_2_ and photo-controlled reversible conversion of supramolecular assemblies based on water soluble pillar[5]arene and coumarin-containing guest. Chin. Chem. Lett..

[23] Zhang W., Gong X., Liu C., Piao Y., Sun Y., Diao G. (2014). Water-soluble inclusion complex of fullerene with γ-cyclodextrin polymer for photodynamic therapy. J. Mater. Chem. B.

[24] Celebioglu A., Uyar T. (2020). Fast-dissolving antioxidant curcumin/cyclodextrin inclusion complex electrospun nanofibrous webs. Food Chem..

[25] Zhang W., Yang J., Li X., Chen T., Park S., Bae M., Jung D., Lin L., Paek S.H., Piao Y. (2022). Anticancer pH-Responsive Supramolecular Vesicles Fabricated Using Water-Soluble Pillar[5]arene and Curcumin Derivative. Mater. Des..

